# Prognostic significance and multidimensional roles of interferon regulatory factors in cancer biology: A comprehensive analysis

**DOI:** 10.1016/j.gendis.2024.101426

**Published:** 2024-09-07

**Authors:** Guo Ji, Shuyuan Xian, Jiaqi Song, Shiyu Mao, Man Pi, Qiongyi Huang, Xue Han, Jian Yin, Zan Wu, Runzhi Huang, Dongyan Han, Zhengyan Chang

**Affiliations:** a Department of Pathology, Shanghai Tenth People's Hospital, School of Medicine, Tongji University, Shanghai 200072, China; b Department of Burn Surgery, The First Affiliated Hospital of Naval Medical University, Shanghai 200433, China; c Shanghai Jiao Tong University School of Medicine, Shanghai 200025, China; d Department of Urology, Shanghai Tenth People's Hospital, School of Medicine, Tongji University, Shanghai 200072, China; e Department of General Surgery, General Hospital of Administration Bureau for Petrolium of North China, Hebei 062552, China; f Department of Orthopedics, Shibei Hospital, Shanghai, 200443, China

Interferon regulatory factors (IRFs) are transcription factors with a conserved N-terminal helix-loop-helix DNA-binding domain ^1^. IRF family plays a pivotal role in regulating interferon transcription, immune cell development, cell growth, apoptosis, and oncogenesis.^2^ Despite considerable research, the roles of IRFs in cancer development, metastasis, drug resistance, and prognosis remain unclear. Through multidimensional correlation analysis, we examined the association between IRFs and various cancer characteristics, including clinical and immune subtype analysis, stemness, tumor microenvironment (TME), and drug sensitivity, by utilizing The Cancer Genome Atlas (TCGA) data. Moreover, the classification and prognostic role of IRF1 were further validated through immunohistochemistry staining of kidney renal papillary cell carcinoma (KIRP) clinical tissues. This research enriches our understanding of the IRFs' roles in cancer and their clinical applications.

Data on the IRFs family across 33 TCGA cancer types (comprising 11,057 adjacent tissues and tumor samples) were downloaded from the UCSC Xena database (http://xena.ucsc.edu/), with cancer types listed in Table S1 and the workflow in Figure S1. Differential expression analysis using the R package "ggpubr" (Wilcoxon test) showed significant IRFs gene regulation in various cancers compared with adjacent tissues (Fig. S2A–M). Using RNA sequencing and reverse-phase protein array data, the LinkedOmics database (http://www.linkedomics.org/login.php) identified proteins associated with IRF1, IRF2, IRF3, and IRF5 in TCGA cancers. Pearson's R analysis results were represented in volcano plots (Fig. S3A–H), which includes analyses for IRF1 in breast invasive carcinoma, esophageal carcinoma (ESCA), and lung adenocarcinoma (LUAD), IRF2 in breast invasive carcinoma and esophageal carcinoma, IRF3 in breast invasive carcinoma and kidney renal clear cell carcinoma, and IRF5 in brain lower grade glioma. Proteins with significant correlations with the IRF genes are highlighted, indicating potential key players in the IRF-related pathways in different cancers.

Patients were categorized into low- and high-expression groups based on median IRF expression levels, using phenotype and survival data from Genomic Data Commons TCGA datasets. Kaplan–Meier analyses of 13 IRFs across TCGA cancers revealed a broad association between IRF gene expression and prognosis (Table S2 and Fig. 1A–I). High IRF1 expression correlated with poor prognosis in KIRP and brain lower-grade glioma but better prognosis in skin cutaneous melanoma. Low IRF2 levels were favorable in acute myeloid leukemia. Elevated IRF3, IRF7, and IRF9 expression were associated with worse prognosis in kidney renal clear cell carcinoma, whereas higher IRF6 levels indicated better outcomes. High IRF5 expression was associated with adverse prognosis in brain lower-grade glioma. Cox regression confirmed the prognostic value of IRF genes across cancers, with a forest plot (Fig. 1J).

**Figure 1:**
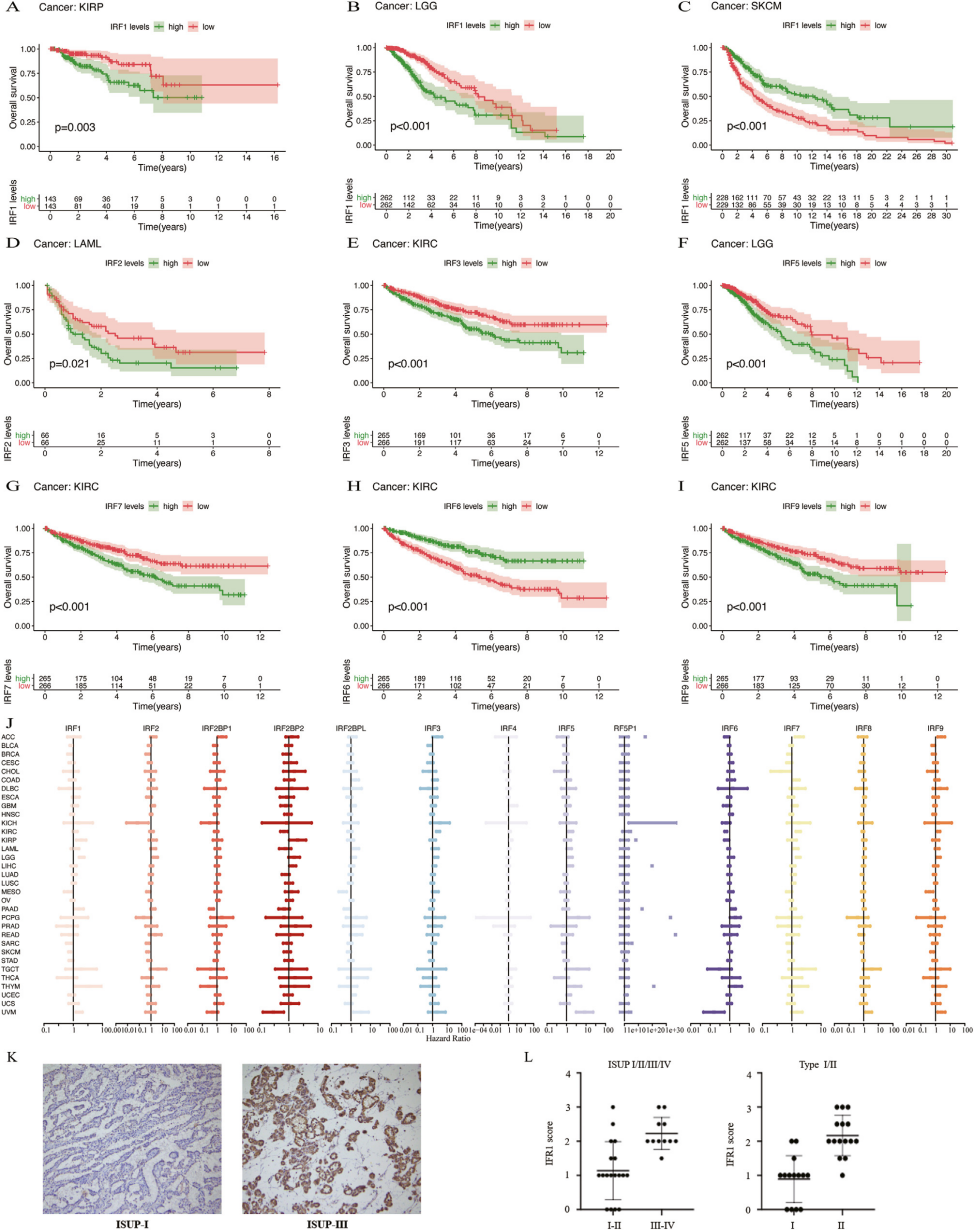
Analysis of survival, drug sensitivity, and immunohistochemistry staining for the IRF family. **(A**–**I)** Kaplan–Meier survival curves display the overall survival for different expression groups of the IRF family genes. **(J)** A forest plot illustrates the IRF family genes as risk or protective factors based on Cox regression analysis. **(K)** Immunohistochemistry staining comparisons between the low and high ISUP grade groups in KIRP cases. **(L)** Scores for IRF1 were significantly higher in the high ISUP group and histology type Ⅱ compared with the low ISUP group and histology type Ⅰ, respectively.

The tumor immune microenvironment in anti-tumor therapies has prognostic and therapeutic significance. The distribution of immune subtypes has different biological and clinical features. Some studies suggest that interferon-γ, induced by IRFs, may promote tumor progression by acting together as immunosuppressive mediators in the tumor microenvironment. Six immune subtypes were identified in the TCGA tumors: C1 (wound healing), C2 (interferon-γ dominant), C3 (inflammatory), C4 (lymphocyte depleted), C5 (immunologically quiet), and C6 (transforming growth factor-β dominant). Using the Kruskal–Wallis test, we analyzed the expression of 13 IRFs across these subtypes. Most IRFs showed higher expression in C1, C2, and C3 than in C4, C5, and C6, with the highest expression in C2, suggesting a regulatory relationship between IRFs and tumor microenvironment (Fig. S4A). Similar patterns were observed in liver hepatocellular carcinoma (Fig. S4B), lung adenocarcinoma (Fig. S4C), sarcoma (Fig. S4D), and skin cutaneous melanoma (Fig. S4E). These results might reveal an underlying correlation between IRF family and six tumor immune subtypes.

The stromal and immune scores and tumor purity were calculated using the ESTIMATE (Estimation of STromal and Immune cells in MAlignant Tumors using Expression data) algorithm.^3^ These analyses demonstrated broad correlations of most IRFs with these metrics across 33 cancer types (Fig. S5). IRF1, IRF2, IRF4, IRF5, IRF7, and IRF8 were positively correlated with the stromal and immune scores and negatively correlated with tumor purity (Fig. S5A–C). Furthermore, stemness was proposed to describe the self-renewal and dedifferentiation of the stem-cell-like characteristics of tumors.^4^ Two stemness indices, namely DNAss (DNA methylation-based stemness index) and RNAss (mRNA expression-based stemness index) were analyzed. Substantial correlations were discovered, with most IRFs showing a negative correlation with DNAss in KIRP, brain lower grade glioma, and uveal melanoma, and a positive correlation with RNAss, suggesting intricate relationships between IRFs and stemness indices (Fig. S5D, E). These associations were consistent across individual cancer types (Fig. S6A–D).

Drug activity data and RNA sequencing profiles for IRF genes were extracted from CellMiner (https://discover.nci.nih.gov/cellminer/).^5^ Pearson correlation analysis detected the association between IRFs and compound sensitivity. IRF gene expression levels were correlated with drug sensitivity, as demonstrated in scatter plots (Fig. S7). Significant correlations, defined by *P* < 0.05, indicated that IRF4 had a strong positive correlation with several compounds, including vemurafenib (*r* = 0.62), denileukin diftitox (Ontak) (*r* = 0.62), dabrafenib (*r* = 0.60), hypothemycin (*r* = 0.49), bafetinib (*r* = 0.42), and selumetinib (*r* = 0.42). However, IRF4 showed resistance to dasatinib (*r* = −0.44) and irofulven (*r* = −0.41). Similar strong correlations were observed for other IRFs (Table S3). Some IRFs were negatively correlated with drug sensitivity, suggesting a role in drug resistance across various cancers. For instance, IRF1 was significantly associated with decreased sensitivity to bafetinib (*r* = −0.366), cobimetinib (*r* = −0.339), dabrafenib (*r* = −0.341), and vemurafenib (*r* = −0.345), with *P* < 0.01 (Table S3).

Given the broad and significant correlation of IRF1 with our multidimensional analyses, the immunohistochemical staining of specimens was performed to validate these findings from 29 patients with KIRP. The study by experienced pathologists involved determining the percentage of IRF1-positive tumor cells and evaluating the histochemical score. The 29 KIRP samples were categorized into two groups (15 in the low group and 14 in the high group) based on the histochemical gene score of IRF1. Further analysis using the International Society of Urological Pathology (ISUP) grading system revealed a significant association between higher IRF1 expression levels and higher ISUP grades, indicative of poorer prognosis (Fig. 1K, L). No significant differences were observed in baseline characteristics (age, gender, and tumor location), tumor volume, and Ki-67 labeling between the two groups (Table S4). Moreover, the histochemical gene scores for IRF1 in the high ISUP group and histology type II were substantially higher than in the low ISUP group and histology type I (Fig. 1L), respectively (Table S4). These immunohistochemistry findings explore the prognostic significance of IRF1 in KIRP, demonstrating a positive correlation between IRF1 protein levels and higher ISUP grades and histology type, suggesting adverse overall survival outcomes.

In summary, the multidimensional exploration of the role of IRFs established them as efficient biomarkers for cancer diagnosis and prognosis and highlighted their potential as targets for therapeutic intervention. Our findings deepen the understanding of the intricate interplay between IRFs and cancer, offering valuable insights for future clinical practice and therapeutic development. Despite its contributions, this study acknowledges limitations, such as the retrospective nature of our analyses and potential biases inherent in using publicly available datasets. Moreover, the complex regulatory networks involving IRFs necessitate further mechanistic studies to elucidate the precise molecular mechanisms underlying their diverse roles in cancer. These limitations highlight the necessity for future prospective research and functional experiments to validate our findings and unravel the intricate dynamics by which IRFs influence carcinogenesis. Despite these constraints, our study lays the groundwork for subsequent in-depth investigations into the clinical implications of the IRF family in diverse cancer scenarios.

## Ethics declaration

This study was approved by the Ethics Committee of Shanghai Tenth People's Hospital (No. 22KN104).

## Funding

This study was supported in part by the 10.13039/501100001809National Natural Science Foundation of China (No. 82002923, 82203505).

## Data availability

The datasets generated and/or analysed during the current study are available in the TCGA program (https://portal.gdc.cancer.gov).

## Author contributions

All authors collaboratively contributed to the work throughout the whole process, including Conceptualization, Data curation, Formal analysis, Investigation, Methodology, Project administration, Resources, Software, Supervision, Validation, Visualization, Writing – original draft. All authors read and approved the final published version.

## Conflict of interests

The authors declared that the research was conducted in the absence of any commercial or financial relationships that could be construed as a potential conflict of interest.
