# Supplementary material for: Prognostic significance and multidimensional roles of interferon regulatory factors in cancer biology: A comprehensive analysis
Source: Genes Dis. 2024 Sep 7;12(3):101426. doi: 10.1016/j.gendis.2024.101426 (PMC11786875; doi:10.1016/j.gendis.2024.101426)
Supplement: Multimedia component 1 [file mmc1.docx]

## Supplementary Materials and Methods

## Materials and Methods

**Data downloading**

On June 26, 2020, data for IRF1, IRF2, IRF2BP1, IRF2BP2, IRF2BPL, IRF3, IRF4, IRF5, IRF5P1, IRF6, IRF7, IRF8, IRF9 in 33 types of TCGA cancers (11 057 adjacent tissues and tumor samples) were downloaded from UCSC Xena database (<http://xena.ucsc.edu/>). Related demographics, tumor information and survival data were extracted. The TCGA cancer types and abbreviations are detailed in Table S1.

**Differential expression analysis and co-expression of IRF genes**

The R package “ggpubr” was used to calculate differential expression analysis (Wilcox test). Differences were showed in a heatmap with the form of log2 Fold Change (log2 FC). The co-expression pattern between two IRF genes was explored using the R package “corrplot”.

**Survival analysis**

Patients were divided into low- and high-expression groups based on the median expression level of IRFs, using phenotype and survival data from the GDC TCGA sets. Kaplan-Meier plots were generated using the R package to show differences in overall survival outcomes in the two groups. Cox proportional hazard regression was used to calculate the Hazard Ratios of IRFs in cancer, and IRF expression in different stages of specific cancer was explored using differential analysis.

**Immune subtype analysis**

The immune TME in anti-tumor therapies has prognostic and therapeutic significance. The distribution of immune subtypes has different biological and clinical features.Six immune subtypes were identified in the TCGA tumor types, including: C1 (wound healing), C2 (IFN-γ dominant), C3 (inflammatory), C4 (lymphocyte depleted), C5 (immunologically quiet) and C6 (TGF-β dominant). We performed differential expression analysis to explore expression levels in 13 IRFs in these subtypes, using the Kruskal test.

**TME and Stemness Indices in cancer**

ESTIMATE evaluates the proportion of TME components (stromal and immune cells) in malignancies, using expression data. The ESTIMATE score, which reflects tumor purity accurately, was obtained based on gene expression signatures.

The stemness indices of TCGA tumor samples were calculated using the one-class logistic regression algorithm and the Spearman correlation analysis (based on stemness scores and gene expression). Two kinds of stemness indices were obtained, namely DNAss (DNA methylation-based stemness index) and RNAss (mRNA expression-based stemness index).

We explored the correlation relationship between the transcriptional expression of IRFs and RNAss and DNAss by calculating the Stromal, Immune and ESTIMATE scores (the algebraic sum of Stromal score and Immune score) in BRCA, COAD, HNSC and LIHC.

**Drug sensitivity analysis**

Drug activity data and data for the RNA-seq profiles for IRF genes were extracted from CellMiner (<https://discover.nci.nih.gov/cellminer/>), which is a web-based tool that provides pharmacologic and genomic information regarding drug responses and transcript data in the NCI-60 cell line sets. Raw data was pre-processed using the “Impute” R package from Bioconductor and the Pearson correlation analysis was used to detect the association between the IRFs and compound sensitivity.

**Validation with immunohistochemistry staining**

We further explored the correlation between IRFs and tumor prognosis. We collected pathology samples from 29 KIRP patients. We assessed the immunohistochemistry staining of samples, advised by experienced pathologies, to determine the percentage of IRF1 positive tumor cells, and evaluate the histochemistry score. This considers the intensity score and the percentage of cells with that intensity.

**Statistic and software.**

All data were processed by R 3.6. Two-sided P < 0.05 was defined as statistically significant for all statistical tests above.

**Supplementary Table and figure legends**

**Table S1 33 TCGA cancer types and their abbreviations.**

| Cancer | Abbreviation | Cancer | Abbreviation |
| --- | --- | --- | --- |
| Adrenocortical carcinoma | ACC | Lung adenocarcinoma | LUAD |
| Bladder Urothelial Carcinoma | BLCA | Lung squamous cell carcinoma | LUSC |
| Breast invasive carcinoma | BRCA | Mesothelioma | MESO |
| Cervical squamous cell carcinoma and endocervical adenocarcinoma | CESC | Ovarian serous cystadenocarcinoma | OV |
| Cholangiocarcinoma | CHOL | Pancreatic adenocarcinoma | PAAD |
| Colon adenocarcinoma | COAD | Pheochromocytoma and Paraganglioma | PCPG |
| Lymphoid Neoplasm Diffuse Large B-cell Lymphoma | DLBC | Prostate adenocarcinoma | PRAD |
| Esophageal carcinoma | ESCA | Rectum adenocarcinoma | READ |
| Glioblastoma multiforme | GBM | Sarcoma | SARC |
| Head and Neck squamous cell carcinoma | HNSC | Skin Cutaneous Melanoma | SKCM |
| Kidney Chromophobe | KICH | Stomach adenocarcinoma | STAD |
| Kidney renal clear cell carcinoma | KIRC | Testicular Germ Cell Tumors | TGCT |
| Kidney renal papillary cell carcinoma | KIRP | Thyroid carcinoma | THCA |
| Acute Myeloid Leukemia | LAML | Thymoma | THYM |
| Brain Lower Grade Glioma | LGG | Uterine Corpus Endometrial Carcinoma | UCEC |
| Liver hepatocellular carcinoma | LIHC | Uterine Carcinosarcoma | UCS |
| Uveal Melanoma | UVM |  |  |

**Table S2** The HR of Cox proportional hazards regression analysis of IRF family genes in 33 TCGA cancers.

| Cancer | IRF1 | IRF2 | IRF2BP1 | IRF2BP2 | IRF2BPL | IRF3 | IRF4 | IRF5 | IRF5P1 | IRF6 | IRF7 | IRF8 | IRF9 |
| --- | --- | --- | --- | --- | --- | --- | --- | --- | --- | --- | --- | --- | --- |
| ACC | 1.04 | 1.58 | 2.22* | 1.27 | 1.19 | 2.11 | 0.47 | 0.63 | 8.30E+14 | 0.71* | 1.80*** | 0.84 | 2.47** |
| BLCA | 0.86* | 0.74* | 0.75 | 0.98 | 0.85 | 0.62*** | 0.82 | 0.81* | 0.00 | 0.93 | 0.95 | 0.97 | 0.71** |
| BRCA | 0.77** | 0.51*** | 0.78* | 0.86 | 0.96 | 0.88 | 0.84 | 0.95 | 6.23 | 0.99 | 0.83* | 0.92 | 0.76* |
| CESC | 0.88 | 0.79 | 0.96 | 1.13 | 0.92 | 0.71 | 0.67* | 0.85 | 0.00 | 1.18 | 0.82 | 0.80 | 0.98 |
| CHOL | 0.83 | 0.90 | 1.04 | 1.53 | 0.93 | 0.62 | 0.64 | 0.53 | 0.00 | 1.10 | 0.42* | 0.75 | 0.60 |
| COAD | 0.92 | 1.09 | 1.18 | 1.03 | 1.64* | 1.46 | 0.80 | 1.18 | 0.01 | 1.16 | 1.43** | 1.05 | 1.24 |
| DLBC | 0.74 | 1.29 | 0.74 | 1.25 | 1.21 | 0.54 | 0.92 | 1.27 | 0.00 | 1.43 | 1.58 | 0.65 | 2.02 |
| ESCA | 1.21 | 0.68 | 0.92 | 0.86 | 0.83 | 1.26 | 0.89 | 0.92 | 0.07 | 0.93 | 0.93 | 1.15 | 1.02 |
| GBM | 1.12 | 1.42 | 1.04 | 0.79 | 1.28 | 1.14 | 3.24 | 1.20 | 0.00 | 0.73 | 1.26* | 0.99 | 0.92 |
| HNSC | 0.91 | 1.03 | 0.79 | 0.93 | 0.91 | 0.93 | 0.71*** | 0.93 | 0.08 | 1.16 | 0.96 | 0.80** | 0.81 |
| KICH | 1.48 | 0.07* | 0.32 | 0.85 | 0.98 | 3.19 | 0.56 | 1.20 | 6.27E+98** | 0.66 | 1.70 | 1.29 | 1.51 |
| KIRC | 1.15 | 1.11 | 0.89 | 0.68** | 0.89 | 2.38*** | 1.40** | 1.63*** | 2.20E+04 | 0.63*** | 1.65*** | 0.89 | 1.65*** |
| KIRP | 1.86** | 1.41 | 1.08 | 2.00* | 0.96 | 1.03 | 1.66 | 1.14 | 1.68E+08 | 0.69* | 1.02 | 1.10 | 1.03 |
| LAML | 1.14 | 1.56 | 1.07 | 0.75 | 1.81*** | 1.51 | 1.15 | 1.36* | 0.63 | 0.89 | 1.33* | 1.18* | 1.29 |
| LGG | 1.94*** | 2.38*** | 1.22 | 1.52 | 1.34* | 1.80*** | 0.90 | 1.58*** | 0.03 | 1.16 | 1.65*** | 1.11 | 1.62*** |
| LIHC | 1.03 | 1.11 | 0.99 | 1.36* | 1.10 | 1.32* | 0.89 | 1.53** | 6.73E+19 | 0.93 | 1.09 | 0.98 | 1.03 |
| LUAD | 1.08 | 0.97 | 1.23 | 0.78 | 0.98 | 1.06 | 0.79** | 0.88 | 0.01 | 0.96 | 1.13 | 0.78** | 0.85 |
| LUSC | 1.04 | 0.94 | 0.96 | 1.02 | 0.93 | 0.97 | 1.12 | 0.97 | 5.13 | 0.96 | 1.13 | 1.02 | 1.01 |
| MESO | 0.67* | 0.59 | 1.15 | 1.25 | 0.38*** | 1.55 | 0.92 | 0.84 | 23.34 | 1.14 | 0.81 | 0.91 | 0.39** |
| OV | 0.95 | 0.88** | 1.15 | 1.05 | 1.08 | 0.99 | 0.71* | 0.99 | 3.42 | 0.86 | 0.96 | 0.94 | 0.90 |
| PAAD | 1.55* | 1.46 | 0.42** | 0.69 | 0.61** | 0.85 | 0.88 | 0.92 | 4.71E+12 | 1.52** | 1.11 | 1.10 | 1.06 |
| PCPG | 1.10 | 0.23 | 2.13 | 0.68 | 1.87 | 1.45 | 0.00 | 4.06* | 1.03E+33 | 1.94* | 0.81 | 0.39 | 0.46 |
| PRAD | 0.61 | 1.07 | 1.12 | 1.72 | 1.30 | 1.44 | 0.29 | 0.71 | 0.00 | 1.10 | 0.78 | 1.16 | 0.92 |
| READ | 0.74 | 2.31 | 1.02 | 1.06 | 1.86 | 1.00 | 0.79 | 1.11 | 4.30E+36 | 1.12 | 1.08 | 0.69 | 0.90 |
| SARC | 0.72** | 0.52*** | 1.01 | 0.89 | 0.89 | 1.21 | 0.91 | 0.77* | 2.24E+05 | 0.92 | 0.84 | 0.81* | 0.89 |
| SKCM | 0.76*** | 0.52*** | 1.06 | 0.89 | 0.81 | 0.71* | 1.06 | 0.78** | 9.38 | 1.11 | 0.83** | 0.80*** | 0.58*** |
| STAD | 0.85 | 0.74 | 0.81 | 0.98 | 1.01 | 0.86 | 0.92 | 0.97 | 3.53 | 0.94 | 0.96 | 0.96 | 0.88 |
| TGCT | 1.85 | 4.03 | 0.37 | 1.20 | 1.58 | 0.88 | 2.30 | 3.62 | 0.00 | 0.30 | 2.25 | 3.61 | 2.06 |
| THCA | 0.61 | 0.46 | 1.24 | 1.64 | 2.18 | 0.63 | 1.22 | 0.56 | 0.00 | 1.18 | 1.02 | 1.18 | 0.47 |
| THYM | 3.36* | 1.41 | 0.27* | 1.59 | 0.72 | 0.64 | 1.05 | 2.57* | 1.19E+21* | 1.96 | 1.74 | 1.22 | 1.80 |
| UCEC | 0.88 | 0.68* | 1.11 | 0.95 | 1.41** | 0.97 | 0.76 | 1.30* | 17.14* | 0.88 | 0.92 | 0.99 | 1.41* |
| UCS | 1.03 | 1.15 | 1.35 | 1.09 | 0.77 | 1.34 | 0.91 | 0.83 | 0.00 | 0.97 | 0.93 | 0.98 | 1.10 |
| UVM | 1.60*** | 0.90 | 0.50 | 0.30** | 3.26* | 1.96 | 0.99 | 8.47*** | 3.15E+04 | 0.16** | 1.48 | 2.20** | 2.53** |

HR: Hazard Ratio, *:P < 0.05, **: P < 0.01, ***: P < 0.001.

Table S3. The significant correlations between drug sensitivity and IRF gene expression

| Gene | Drug | correlation coefficient | | P value |
| --- | --- | --- | --- | --- |
| IRF1 | Tanespimycin | | -0.44 | < 0.001 |
|  | geldanamycin analog | | -0.39 | 0.002 |
|  | Asparaginase | | 0.39 | 0.002 |
|  | Nelarabine | | 0.39 | 0.002 |
|  | Bafetinib | | -0.37 | 0.004 |
|  | Vemurafenib | | -0.35 | 0.007 |
|  | Dabrafenib | | -0.34 | 0.008 |
|  | Cobimetinib (isomer 1) | | -0.34 | 0.008 |
|  | LMP-400 | | 0.33 | 0.010 |
|  | Bendamustine | | 0.32 | 0.013 |
|  | Hydroxyurea | | 0.32 | 0.014 |
|  | Selumetinib | | -0.31 | 0.016 |
|  | Trametinib | | -0.31 | 0.017 |
|  | Clofarabine | | 0.30 | 0.020 |
|  | PD-98059 | | -0.30 | 0.020 |
|  | Chelerythrine | | 0.30 | 0.022 |
|  | Panobinostat | | -0.29 | 0.022 |
|  | Chlorambucil | | 0.29 | 0.026 |
|  | Fludarabine | | 0.28 | 0.029 |
|  | Tyrothricin | | -0.28 | 0.030 |
|  | Tamoxifen | | -0.28 | 0.031 |
|  | Lapachone | | -0.28 | 0.033 |
|  | Alvespimycin | | -0.27 | 0.035 |
|  | Mithramycin | | -0.26 | 0.042 |
|  | Fluphenazine | | 0.26 | 0.043 |
|  | Nilotinib | | -0.26 | 0.044 |
|  | Pyrazoloacridine | | 0.26 | 0.045 |
|  | Triapine | | 0.26 | 0.049 |
|  | AT-13387 | | -0.26 | 0.049 |
| IRF2 | Nelarabine | | 0.38 | 0.003 |
|  | Dasatinib | | -0.37 | 0.003 |
|  | Bortezomib | | 0.36 | 0.004 |
|  | Fluphenazine | | 0.36 | 0.005 |
|  | Irofulven | | -0.36 | 0.005 |
|  | Dabrafenib | | 0.32 | 0.012 |
|  | Sonidegib | | -0.32 | 0.014 |
|  | Bendamustine | | 0.30 | 0.021 |
|  | Wortmannin | | 0.29 | 0.024 |
|  | Crizotinib | | 0.29 | 0.027 |
|  | Estramustine | | 0.28 | 0.029 |
|  | Vemurafenib | | 0.28 | 0.032 |
|  | Aminoflavone | | -0.27 | 0.035 |
|  | Ixazomib citrate | | 0.27 | 0.036 |
|  | PX-316 | | 0.27 | 0.038 |
|  | ABT-199 | | 0.27 | 0.039 |
|  | Cabozantinib | | 0.26 | 0.042 |
|  | Palbociclib | | 0.26 | 0.046 |
|  | Dexamethasone Decadron | | 0.26 | 0.046 |
|  | XL-147 | | 0.26 | 0.047 |
|  | Calusterone | | 0.26 | 0.047 |
|  | Asparaginase | | 0.26 | 0.049 |
| IRF2BP1 | Dasatinib | | -0.41 | 0.001 |
|  | Staurosporine | | -0.38 | 0.003 |
|  | Nelarabine | | 0.37 | 0.004 |
|  | Bosutinib | | -0.30 | 0.018 |
|  | Allopurinol | | -0.29 | 0.025 |
|  | Pipobroman | | 0.28 | 0.027 |
|  | AP-26113 | | -0.28 | 0.030 |
|  | Fenretinide | | 0.27 | 0.035 |
|  | Temsirolimus | | 0.27 | 0.036 |
|  | Cladribine | | 0.27 | 0.036 |
|  | Lificguat | | -0.26 | 0.041 |
|  | Cobimetinib (isomer 1) | | -0.26 | 0.046 |
| IRF2BP2 | Lomustine | | 0.31 | 0.015 |
|  | Fenretinide | | 0.31 | 0.016 |
|  | Megestrol acetate | | 0.31 | 0.017 |
|  | Panobinostat | | -0.30 | 0.019 |
|  | Buthionine sulphoximine | | 0.29 | 0.024 |
|  | Imexon | | 0.29 | 0.027 |
|  | Carmustine | | 0.28 | 0.031 |
|  | Testolactone | | 0.26 | 0.043 |
|  | Itraconazole | | 0.26 | 0.044 |
|  | By-Product of CUDC-305 | | -0.26 | 0.045 |
| IRF2BPL | Everolimus | | 0.38 | 0.003 |
|  | Rapamycin | | 0.33 | 0.010 |
|  | Ponatinib | | -0.33 | 0.010 |
|  | Palbociclib | | -0.31 | 0.017 |
|  | Imexon | | -0.29 | 0.024 |
|  | Amonafide | | -0.28 | 0.033 |
|  | Bosutinib | | -0.27 | 0.034 |
|  | Temsirolimus | | 0.27 | 0.035 |
| IRF3 | Nelfinavir | | 0.33 | 0.010 |
|  | Hydroxyurea | | 0.28 | 0.031 |
|  | 5-fluoro deoxy uridine 10mer | | 0.28 | 0.032 |
| IRF4 | Vemurafenib | | 0.62 | < 0.001 |
|  | Denileukin Diftitox Ontak | | 0.62 | < 0.001 |
|  | Dabrafenib | | 0.60 | < 0.001 |
|  | Hypothemycin | | 0.49 | < 0.001 |
|  | Dasatinib | | -0.44 | < 0.001 |
|  | Bafetinib | | 0.42 | 0.001 |
|  | Selumetinib | | 0.42 | 0.001 |
|  | Irofulven | | -0.41 | 0.001 |
|  | Midostaurin | | -0.37 | 0.004 |
|  | Estramustine | | 0.36 | 0.005 |
|  | PD-98059 | | 0.36 | 0.005 |
|  | Cobimetinib (isomer 1) | | 0.34 | 0.007 |
|  | Entinostat | | 0.34 | 0.008 |
|  | Isotretinoin | | 0.33 | 0.011 |
|  | Pyrazoloacridine | | -0.33 | 0.011 |
|  | Vorinostat | | 0.31 | 0.015 |
|  | Triciribine phosphate | | -0.31 | 0.017 |
|  | Bortezomib | | 0.31 | 0.018 |
|  | Fluphenazine | | 0.29 | 0.024 |
|  | Pipamperone | | 0.29 | 0.024 |
|  | XL-147 | | 0.29 | 0.025 |
|  | okadaic acid | | 0.28 | 0.028 |
|  | Imiquimod | | 0.28 | 0.029 |
|  | Hydrastinine HCl | | 0.28 | 0.030 |
|  | Ixazomib citrate | | 0.28 | 0.031 |
|  | Lapachone | | 0.28 | 0.033 |
|  | Ethinyl estradiol | | 0.27 | 0.034 |
|  | 8-Chloro-adenosine | | -0.27 | 0.034 |
|  | Tamoxifen | | 0.27 | 0.034 |
|  | bisacodyl, active ingredient of viraplex | | -0.27 | 0.039 |
|  | Sonidegib | | -0.27 | 0.040 |
|  | Batracylin | | -0.26 | 0.042 |
|  | Tanespimycin | | 0.26 | 0.047 |
| IRF5 | Denileukin Diftitox Ontak | | 0.57 | < 0.001 |
|  | Megestrol acetate | | 0.47 | < 0.001 |
|  | Isotretinoin | | 0.41 | 0.001 |
|  | Nelfinavir | | 0.40 | 0.002 |
|  | RH1 | | 0.35 | 0.006 |
|  | Alectinib | | 0.34 | 0.009 |
|  | Bleomycin | | 0.33 | 0.009 |
|  | Nitrogen mustard | | 0.33 | 0.010 |
|  | Hydroxyurea | | 0.32 | 0.013 |
|  | 7-Hydroxystaurosporine | | 0.31 | 0.014 |
|  | Temsirolimus | | 0.31 | 0.014 |
|  | O-6-Benzylguanine | | 0.31 | 0.018 |
|  | LDK-378 | | 0.30 | 0.022 |
|  | Everolimus | | 0.29 | 0.026 |
|  | Entinostat | | 0.29 | 0.026 |
|  | Estramustine | | 0.28 | 0.027 |
|  | Idarubicin | | 0.28 | 0.030 |
|  | Raltitrexed | | 0.28 | 0.030 |
|  | Itraconazole | | 0.28 | 0.032 |
|  | Abiraterone | | 0.26 | 0.042 |
|  | Seliciclib | | 0.26 | 0.045 |
|  | Erlotinib | | 0.26 | 0.047 |
|  | LMP-400 | | 0.26 | 0.048 |
| IRF6 | Cisplatin | | -0.43 | 0.001 |
|  | Carboplatin | | -0.41 | 0.001 |
|  | kahalide f | | 0.39 | 0.002 |
|  | Gemcitabine | | -0.39 | 0.002 |
|  | SR16157 | | 0.39 | 0.002 |
|  | bisacodyl, active ingredient of viraplex | | 0.36 | 0.004 |
|  | Bleomycin | | -0.35 | 0.006 |
|  | Uracil mustard | | -0.35 | 0.006 |
|  | Chlorambucil | | -0.34 | 0.008 |
|  | Etoposide | | -0.34 | 0.009 |
|  | Melphalan | | -0.33 | 0.010 |
|  | Carmustine | | -0.33 | 0.011 |
|  | Acetalax | | 0.32 | 0.012 |
|  | By-Product of CUDC-305 | | 0.32 | 0.013 |
|  | Simvastatin | | -0.31 | 0.015 |
|  | Triethylenemelamine | | -0.31 | 0.015 |
|  | Thiotepa | | -0.31 | 0.015 |
|  | Arsenic trioxide | | -0.31 | 0.017 |
|  | Elesclomol | | 0.30 | 0.018 |
|  | Asparaginase | | -0.30 | 0.020 |
|  | Mitoxantrone | | -0.30 | 0.020 |
|  | Raltitrexed | | -0.30 | 0.020 |
|  | LMP-400 | | -0.30 | 0.022 |
|  | Fludarabine | | -0.30 | 0.022 |
|  | Staurosporine | | -0.28 | 0.031 |
|  | Topotecan | | -0.27 | 0.039 |
|  | Zoledronate | | -0.27 | 0.040 |
|  | Tamoxifen | | 0.26 | 0.042 |
|  | tfdu | | -0.26 | 0.042 |
|  | Irinotecan | | -0.26 | 0.045 |
|  | Pipobroman | | -0.26 | 0.049 |
| IRF7 | AP-26113 | | 0.33 | 0.010 |
|  | Sonidegib | | -0.33 | 0.011 |
|  | 8-Chloro-adenosine | | 0.28 | 0.032 |
|  | Itraconazole | | 0.27 | 0.035 |
|  | geldanamycin analog | | -0.27 | 0.040 |
|  | Ixazomib citrate | | 0.26 | 0.042 |
|  | Vismodegib | | 0.26 | 0.045 |
|  | Tyrothricin | | -0.26 | 0.047 |
| IRF8 | Perifosine | | 0.37 | 0.004 |
|  | LDK-378 | | 0.36 | 0.004 |
|  | Isotretinoin | | 0.35 | 0.006 |
|  | Selumetinib | | 0.30 | 0.020 |
|  | Eribulin mesilate | | 0.30 | 0.020 |
|  | Tegafur | | 0.30 | 0.021 |
|  | ABT-199 | | 0.29 | 0.024 |
|  | Simvastatin | | -0.29 | 0.024 |
|  | Erlotinib | | -0.29 | 0.024 |
|  | Ibrutinib | | -0.29 | 0.026 |
|  | Dexrazoxane | | 0.29 | 0.027 |
|  | Parthenolide | | 0.28 | 0.028 |
|  | Palbociclib | | 0.28 | 0.028 |
|  | Cyclophosphamide | | 0.28 | 0.029 |
|  | Imexon | | 0.28 | 0.032 |
|  | Sunitinib | | 0.27 | 0.036 |
|  | Everolimus | | -0.27 | 0.038 |
|  | Nelfinavir | | 0.27 | 0.038 |
|  | Tyrothricin | | 0.27 | 0.039 |
|  | Tamoxifen | | 0.26 | 0.041 |
|  | Lapatinib | | -0.26 | 0.049 |
|  | Rapamycin | | -0.25 | 0.050 |
| IRF9 | By-Product of CUDC-305 | | -0.35 | 0.005 |
|  | geldanamycin analog | | -0.33 | 0.009 |
|  | Nilotinib | | -0.32 | 0.013 |
|  | AT-13387 | | -0.30 | 0.019 |
|  | Itraconazole | | 0.29 | 0.024 |
|  | Simvastatin | | 0.28 | 0.032 |
|  | AFP464 | | -0.26 | 0.046 |
|  | Panobinostat | | -0.26 | 0.047 |

**Table S4** Clinical information and immunohistochemistry staining data for 29 patients with KIRP.

|  | **Low group (15)** | **High group (14)** | **P value** |
| --- | --- | --- | --- |
| Age(years) |  |  | 0.096 |
| <60 | 4 | 8 |  |
| >60 | 11 | 6 |  |
| Gender | 11 | 6 | 0.316 |
| male | 8 | 10 |  |
| female | 7 | 4 |  |
| Tumor Location |  |  | 0.876 |
| Left | 6 | 6 |  |
| Right | 9 | 8 |  |
| ISUP grade |  |  | <0.001 |
| 1-2 | 14 | 4 |  |
| 3 | 1 | 10 |  |
| Type |  |  | <0.001 |
| 1 | 12 | 2 |  |
| 2 | 3 | 12 |  |
| Tumor Volume | 67.97(123.78) | 131.87(186.58) | 0.116 |
| Ki-67 | 0.064 (0.07) | 0.089 (0.08) | 0.137 |
| Gene-Score | 0.833（0.56） | 2.321（0.42） | <0.001 |

KIRP: Kidney renal papillary cell carcinoma; ISUP: International Society of Urological Pathology (ISUP) grading system

**
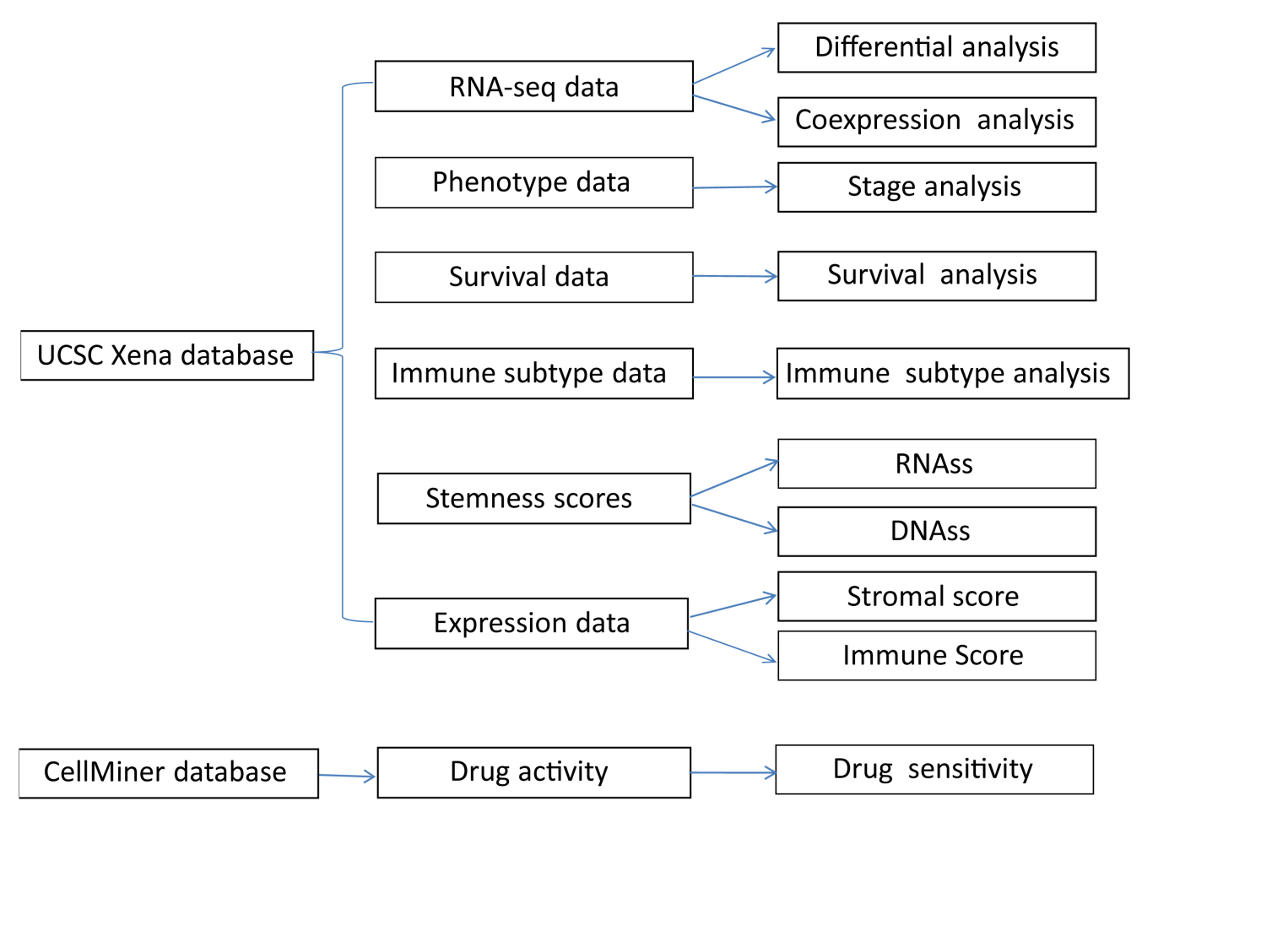
**

**Figure S1.** The flow chart of this study.

**
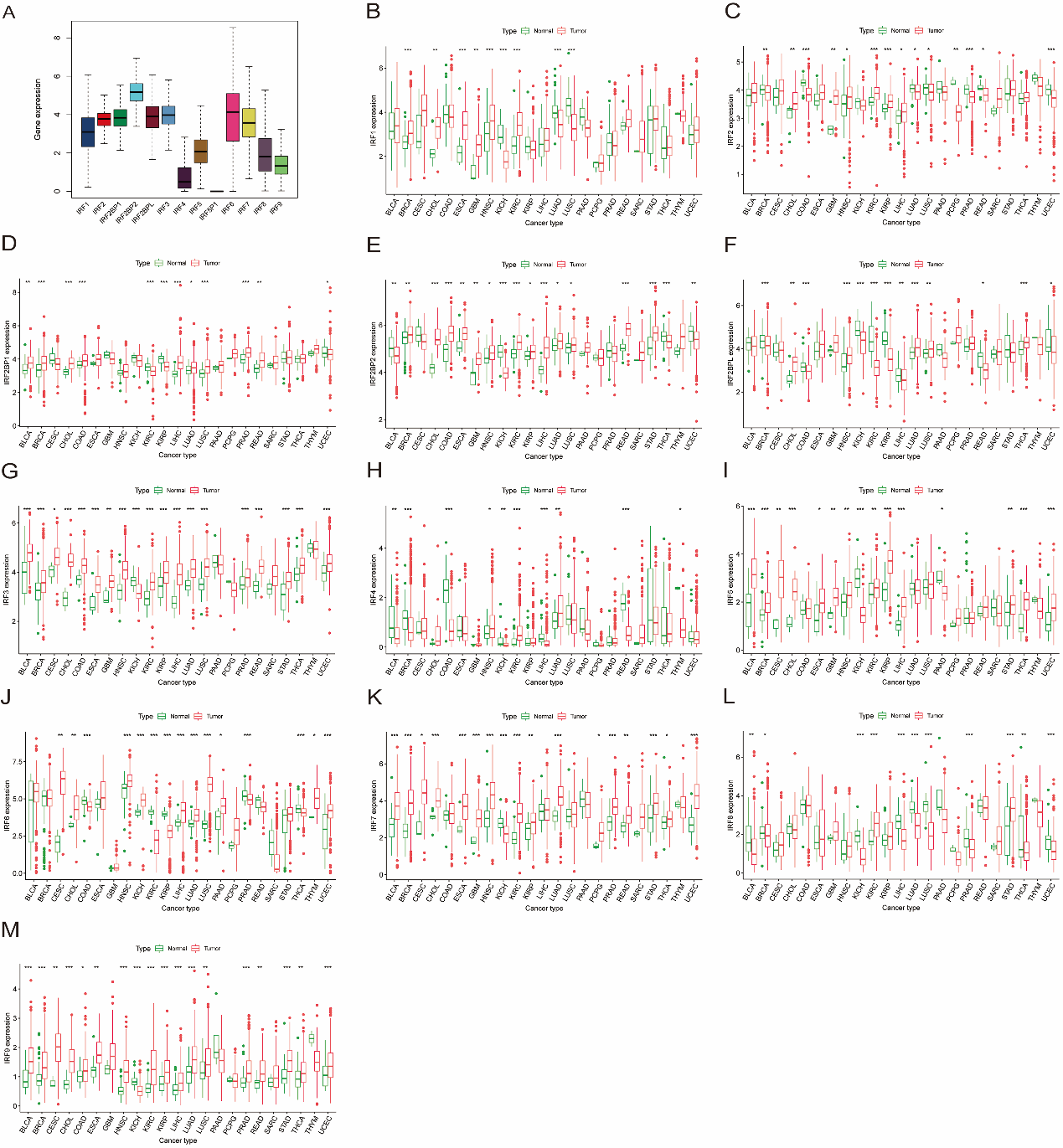
**

**Figure S2.** Expression analysis of IRFs family genes.

(A) A boxplot indicating the median expression levels of *IRF* genes.

(B-M) Differential expression analysis of the *IRF* family between tumor and adjacent samples across various cancers (***: p < 0.001; **: p < 0.01; *: p < 0.05).


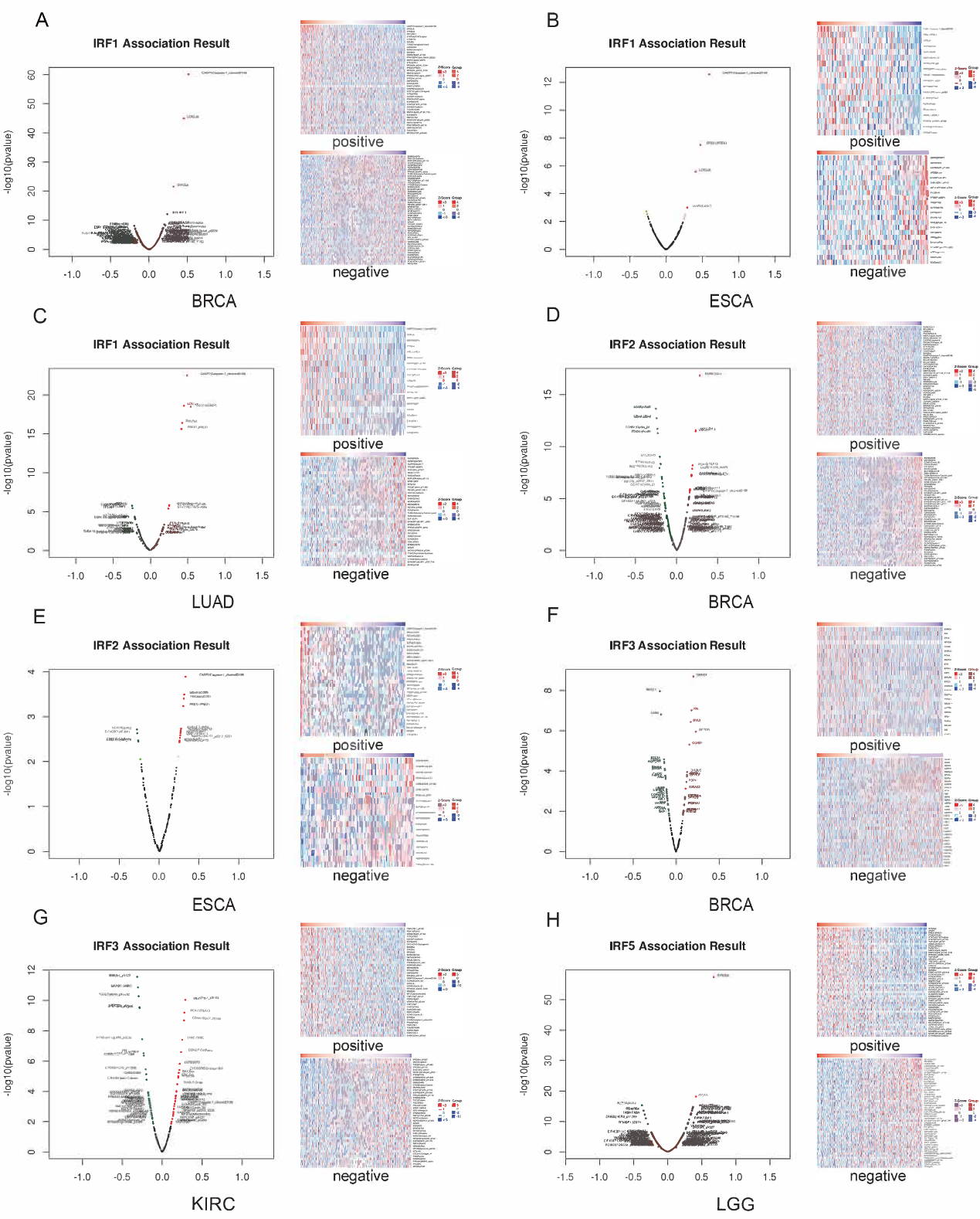


**Figure S3** Volcano plots and a heatmap illustrating proteins positively and negatively correlated with IRFs, based on RPPA data from the LinkedOmics database. This visualization includes analyses for IRF1 in BRCA (A), ESCA (B), and LUAD (C); IRF2 in BRCA (D) and ESCA (E); IRF3 in BRCA (F) and KIRC (G); and IRF5 in LGG (H).

**
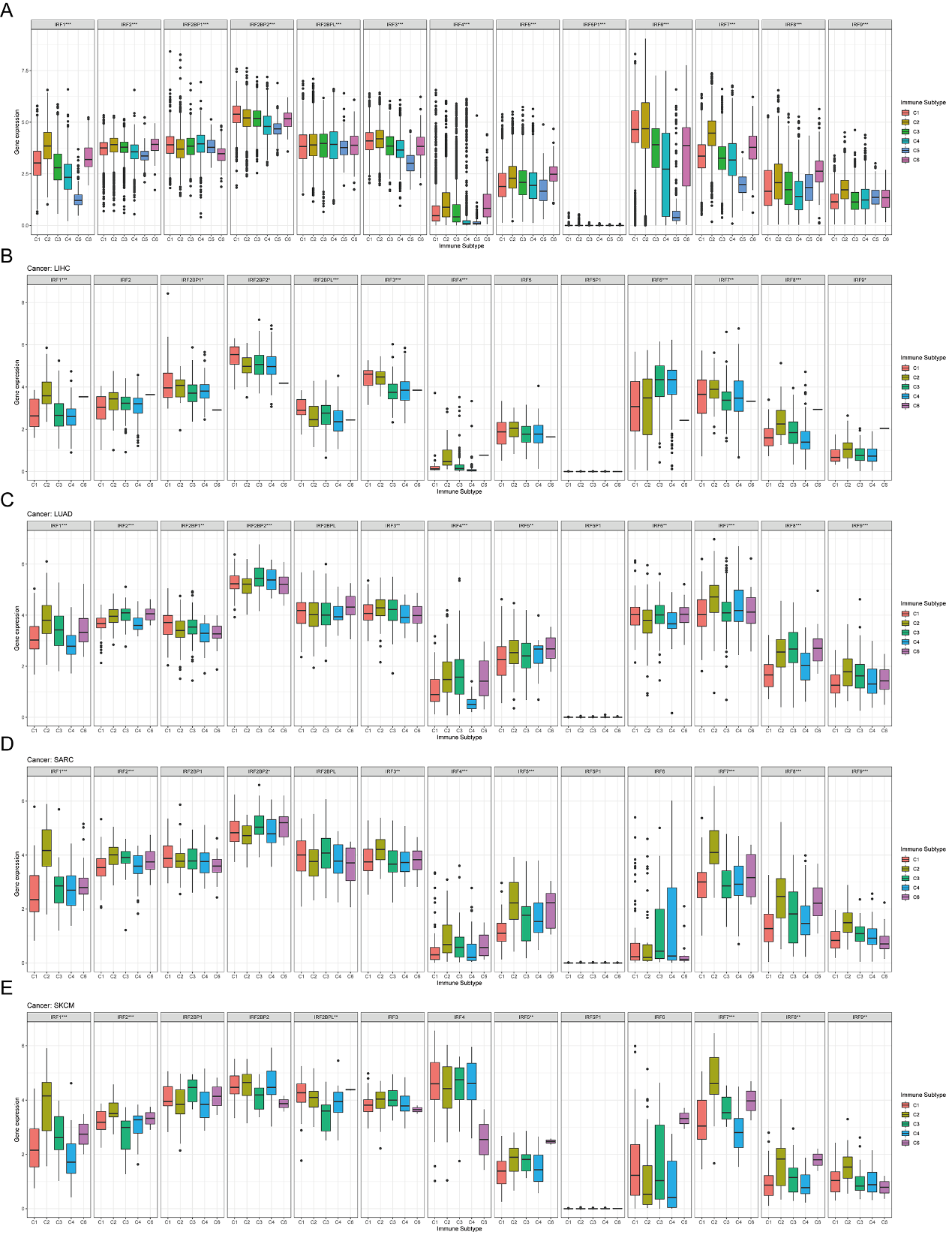
**

**Figure S4.** Correlation analysis between the IRF family and six immune subtypes.

(A) Median expression levels of the IRF family across C1–C6 immune subtypes. Most IRFs showed higher expression in C1, C2, C3 than in C4, C5 and C6, with the highest expression in C2. (B) The expression of IRF1, IRF2, IRF3, IRF4, IRF5 and IRF7, were highest in C2 in LIHC. (C) The expression of IRF1, IRF3, IRF7 and IRF9, were highest in C2 in LUAD. For SARC. (D) The expression of IRF1, IRF2, IRF3, IRF4, IRF5, IRF7, IRF8, and IRF9, were highest in C2 in SARC. (E) The expression of IRF1, IRF2, IRF2BP2, IRF3, IRF5, IRF7, IRF8, and IRF9, were highest in C2 in SKCM. (***: p < 0.001; **: p < 0.01; *: p < 0.05).

**
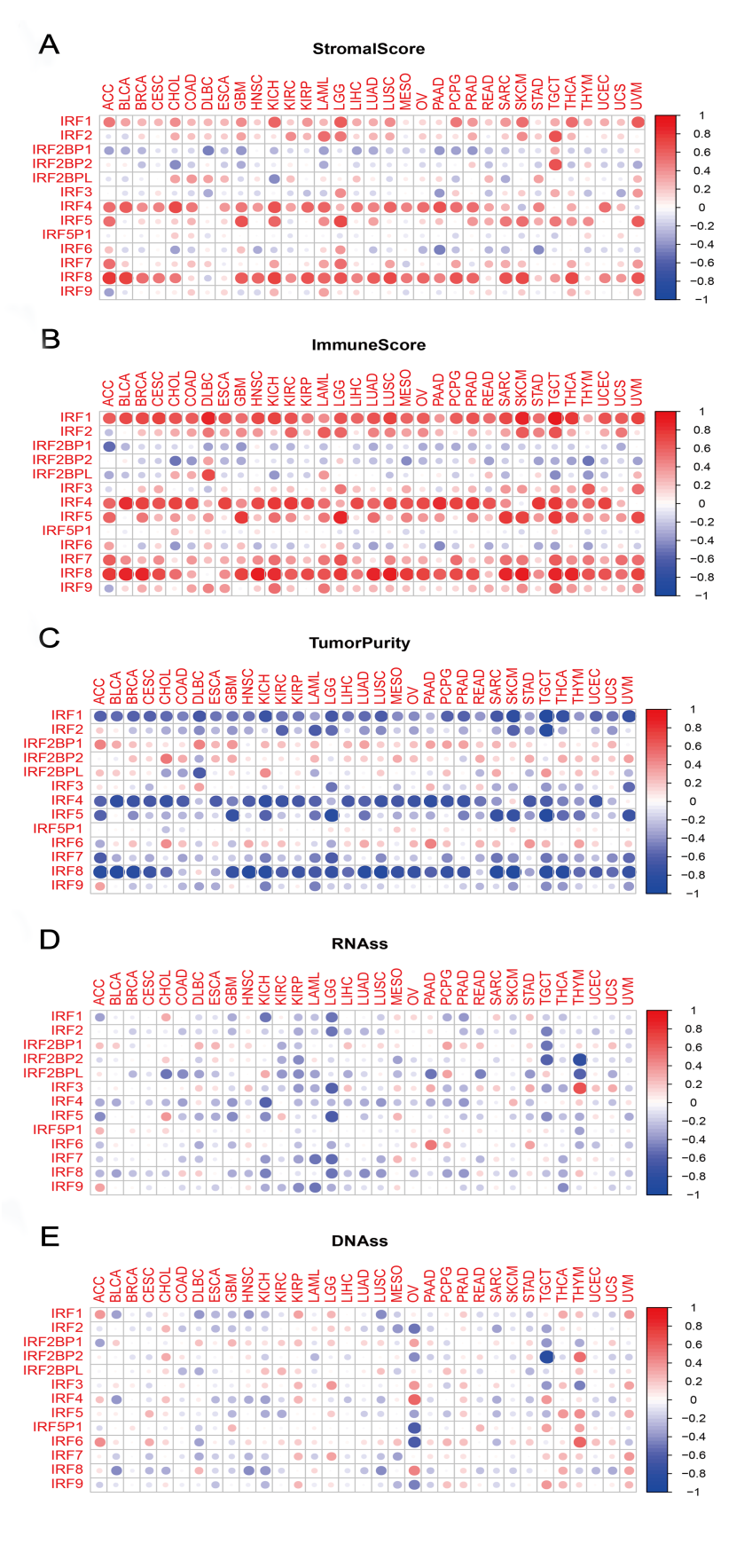
**

**Figure S5.** Correlation analysis between the IRF family and TME.

(A-C) Heatmaps illustrating correlations between the IRF family and stromal scores, immune scores, and tumor purity, alongside RNAss (D) and DNAss (E) correlations (Blue points represent negative correlations while red points represent positive correlations).

**
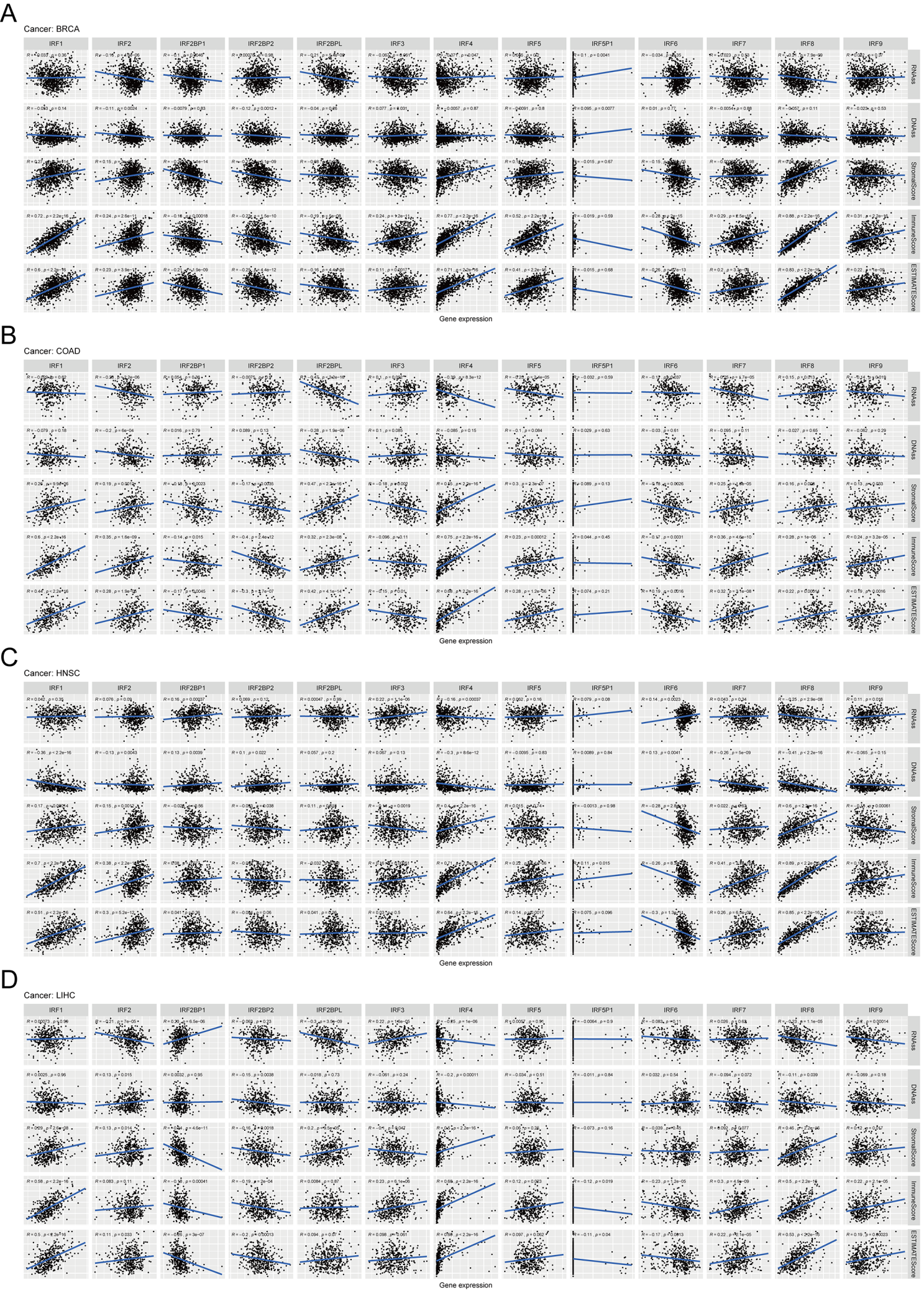
**

**Figure S6.** Specific correlation analysis between individual *IRF* genes, the TME, and stemness indices.

Four examples showing the correlation between IRF family and stemness indices, stromal scores, immune scores, and estimate scores in BRCA (A), COAD (B), HNSC (C), and LIHC (D).

Abbreviations: IRF, Interferon regulatory factor; ISUP, International Society of Urological Pathology; KIRP, kidney renal papillary cell carcinoma; TCGA, The Cancer Genome Atlas; RPPA, reverse-phase protein array; BRCA, breast invasive carcinoma; ESCA, esophageal carcinoma; LUAD, lung adenocarcinoma; KIRC, kidney renal clear cell carcinoma; LGG, brain lower grade glioma; HNSC, head and neck squamous cell carcinoma; COAD, colon adenocarcinoma; LIHC, liver hepatocellular carcinoma; SARC, sarcoma; SKCM, skin cutaneous melanoma; TME, tumor microenvironment.

**
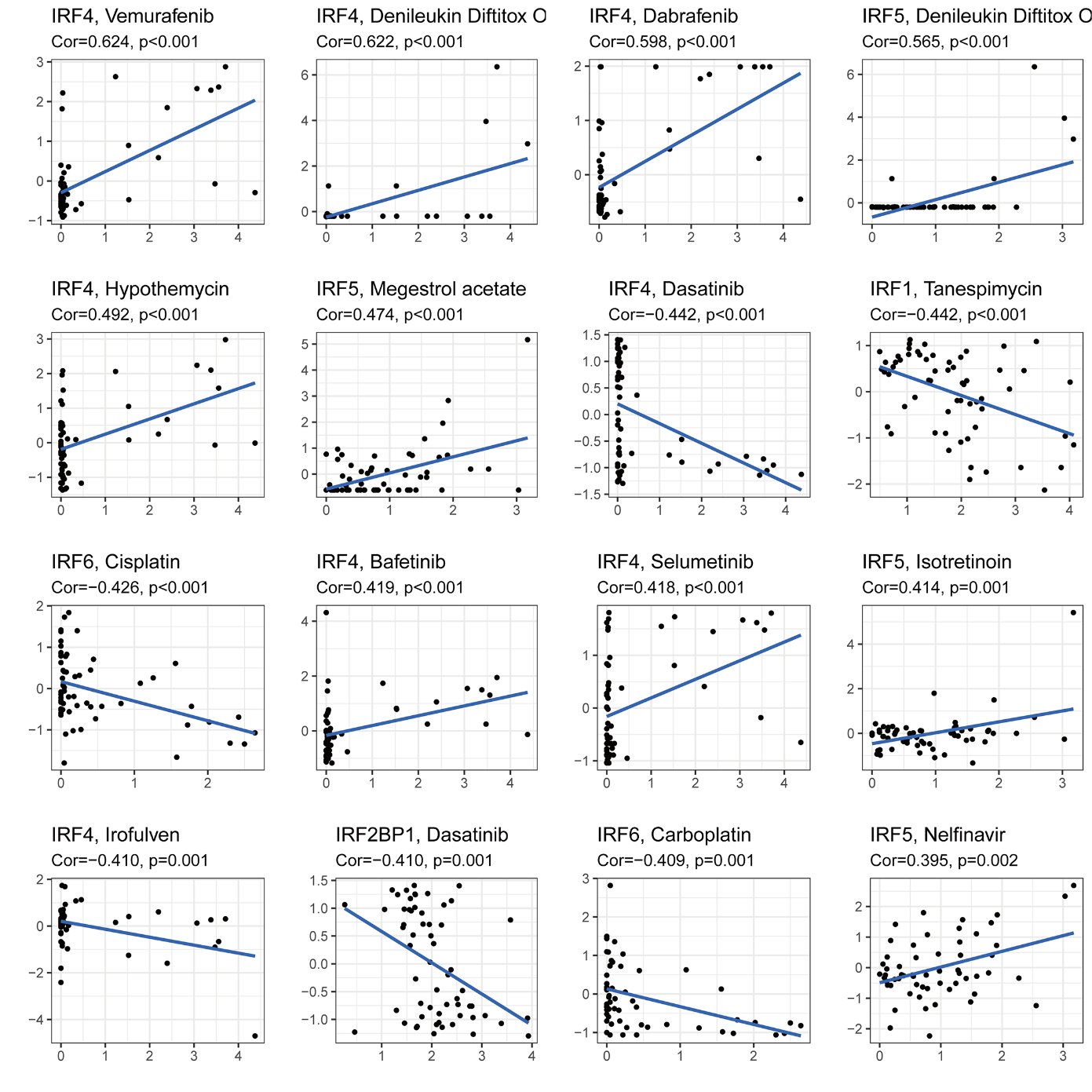
**

**Figure S7.** Scatter plots reveal significant strong correlations between *IRF* family genes and several compounds, including vemurafenib (R = 0.62), denileukin diftitox (Ontak) (R = 0.62), and dabrafenib (R = 0.60).
